# From rationing to rationality: an n-of-one trial service for off-label medicines for rare (neuromuscular) diseases

**DOI:** 10.1186/1750-1172-7-S2-A29

**Published:** 2012-11-22

**Authors:** Stephanie S Weinreich, Charlotte Vrinten, Jan JGM Verschuuren, Carin A Uyl-de Groot, Marja R Kuijpers, Ellen Sterrenburg, Rob JPM Scholten, Cees FRM van Bezooijen, Marcel FTH Timmen, Sonja van Weely, Martina C Cornel

**Affiliations:** 1 Dutch Association for Neuromuscular Disorders, Baarn, 3747 JN, The Netherlands Amsterdam; 2 VU University Medical Center, Amsterdam, 1007 MB, The Netherlands; 3 Leiden University Medical Center, Leiden, 2300 RC, The Netherlands; 4 Dutch Neuromuscular Research Centre, The Hague, 2508 CM, The Netherlands; 5 Erasmus University, Rotterdam, 3000 DR, The Netherlands; 6 Health Care Insurance Board, Diemen, 1110 AH, The Netherlands; 7 Princes Beatrix Fonds, The Hague, 2508 CM, The Netherlands; 8 Dutch Cochrane Centre, Amsterdam, 1100 DD, Amsterdam; 9 Foundation for Equitable Provision of Medicines, Rotterdam, 3062 MB, The Netherlands; 10 former Dutch Steering Committee on Orphan Drugs, The Hague, 2593 CE, The Netherlands

## Background and aims

In the Netherlands, off-label prescription of medicines is tolerated as a suboptimal but inevitable practice. However, patients with rare diseases are disadvantaged in claiming reimbursement for off-label or off-license drugs (here summarised as ‘off-label’). It is difficult to meet the burden of evidence-based proof on efficacy of such medicines. Consensus-based guidelines, a second-best but accepted form of evidence, are also often lacking. Both industry and academia are challenged to perform classical randomized, controlled trials for rare diseases. Moreover, reimbursement rules discourage doctors from prescribing medicines off-label, even to small groups of patients. Thus there is an impasse to creating evidence. Conditionally reimbursed, controlled n-of-one (single-patient) trials with internal randomisation (e.g. AB-BA-BA) could generate evidence on efficacy for rare, chronic conditions where the aim of treatment is symptom control. Practical and scientific support might be provided by a dedicated trial service.

This project aims to initiate development of an n-of-one trial service, embedded in the Dutch health care system, for research on efficacy and safety of certain medicines with no marketing authorisation for the rare diseases for which they are prescribed.

## Methods and preliminary results

Reimbursement problems with off-label medicines were inventoried among neuromuscular specialists and patients with neuromuscular disease. This list was prioritized for medicines and indications suitable for n=1-trials. Two cases were selected for systematic literature review and development of clinical protocols for n-of-one trials. A multidisciplinary expert meeting defined the legal, ethical and scientific preconditions for starting an n-of-one trial service. Recommendations were made for performing n-of-one research in a scientifically sound and socially robust manner.

Regulatory authorities and insurers may accept evidence from n-of-one trials, provided that data can be aggregated and that benefit/risk ratio is considered. An n-of-one trial service may be justified, if it can support a broad range of rare diseases.
